# A manganese metabolism-related gene signature stratifies prognosis and immunotherapy efficacy in kidney cancer

**DOI:** 10.1007/s12672-025-03050-9

**Published:** 2025-07-01

**Authors:** Yang Liu, Hao Ye, Ruoxuan Zhang, Xiaolong Liu, Ranlu Liu

**Affiliations:** Department of Urology, Tianjin Medical University General Hospital, No 154. Anshan Road, Tianjin, 300052 China

**Keywords:** Manganese metabolism, Machine learning, Subtype, Risk signature, RCC

## Abstract

**Background:**

Manganese modulates tumorigenesis and immune regulation. High levels of manganese may promote cancer progression. While manganese toxicity causes renal tubular damage and chronic impairment, its association with kidney cancer remains poorly understood.

**Methods:**

We systematically analyzed manganese metabolism genes in KIRC using the TCGA dataset. Through integrated bioinformatics approaches, including differential expression analysis, univariate Cox regression, and three machine learning algorithms (Boruta, GBM, and RFS), we identified prognosis-related MMCG. The Ward.D2 method was used to identify MMCG subtypes, while Lasso-cox regression analysis was performed to establish the MMCG risk model. The predictive performance was validated through time-dependent ROC analysis, calibration curves, and decision curve analysis.

**Results:**

We identified 11 prognosis-related manganese metabolism core genes (MMCGs). KIRC patients were stratified into two clusters based on MMCG expression levels. Patients in Cluster I showed poorer outcomes, which were associated with tumour progression. The MMCG risk score was subsequently developed using LASSO-Cox regression analysis, and patients were classified into high- and low-risk groups. Survival analysis revealed that the outcomes of high-risk group patients were poorer than those of the low-risk group. Univariate and multivariate analyses confirmed the MMCG risk score as an independent prognostic biomarker. Pathway enrichment analysis showed differential enrichment of immune and metabolic pathways across subtypes and risk groups. We constructed a clinical nomogram incorporating the MMCG risk score and other clinical parameters, which demonstrated highly accurate predictive capabilities. Immune infiltration analysis and immune therapy response predictions indicated that patients in Cluster I and the high-risk group showed low responses to immune therapy.

**Conclusion:**

Our findings provide a basis for clinical stratification strategies and future research on manganese-based interventions for renal cell carcinoma (RCC).

**Supplementary Information:**

The online version contains supplementary material available at 10.1007/s12672-025-03050-9.

## Introduction

Cancer remains a leading global health concern. Renal cell carcinoma (RCC) is emerging as a prevalent malignant neoplasm. In 2020, RCC was responsible for 400,000 new cases and 179,000 fatalities worldwide [1]. Clear cell renal cell carcinoma (KIRC), the most prevalent subtype of RCC, constitutes approximately 75% of all RCC cases [2]. However, there are various existing treatments for KIRC. Unfortunately, about 30% of patients are already in the advanced stage of the tumour at the time of initial diagnosis, which is clearly very unfavourable for the prevention and treatment of KIRC [3]. In comparison, chemotherapy and immunotherapy are standard treatments for KIRC, and the disease's heterogeneity results in variable patient responses [4, 5]. Therefore, there is an urgent need for novel prognostic indicators and stratification strategies for KIRC patients.

Manganese (Mn) is an indispensable micronutrient in human physiology, participating in many physiological processes [6, 7]. Emerging evidence suggests that Mn is pivotal in tumorigenesis and immune system modulation [8]. Manganese ions are primarily hepatically filtered and really excreted, a metabolic pathway involving numerous genes intricately associated with oncogenesis and tumour progression [9, 10]. Studies have demonstrated the utility of manganese metabolism-related genes in predicting survival and treatment outcomes in gastric cancer patients [11]. Manganese is an essential trace element involved in various metabolic pathways and enzyme structures, which plays an important role in the kidneys. Mn-SOD scavenges reactive oxygen species (ROS), protects mitochondria from oxidative stress damage, and maintains kidney health [12]. Manganese also participates in iron metabolism, glucose metabolism, and energy balance, supporting kidney function. Manganese exposure is associated with the progression of chronic kidney disease (CKD), and studies suggest that manganese toxicity may accelerate the transition from CKD to acute kidney injury (AKI) [13]. Manganese induces oxidative stress and increases ROS levels, leading to cellular damage and promoting cancer cell apoptosis [14]. Manganese could damage mitochondria and then affect energy metabolism, promoting the development of renal cancer [15]. Although studies have supported the role of manganese in renal cancer, its mechanisms remain unclear. The correlation between manganese metabolism and renal cancer, particularly KIRC, is not well characterized. Thus, elucidating the role of manganese metabolism in KIRC is both necessary and timely.

This study identifies eleven manganese metabolism core genes (MMCG) in kidney cancer. By developing an MMCG subtype and an MMCG risk signature, we effectively stratify KIRC patients based on prognosis, immune profiles, and treatment responsiveness. Validation across multiple RCC cohorts confirms the robustness of our risk model, highlighting its potential for improving prognostic accuracy and guiding personalized treatment strategies. While prior research has linked manganese metabolism dysfunction to oncogenesis, its role and mechanisms in kidney cancer remain understudied. Our findings highlight the significance of manganese metabolism in RCC, providing a theoretical basis for further experimental validation and target discovery.

## Materials and methods

### Data acquisition

We obtained a dataset encompassing 1399 genes implicated in manganese metabolism from the GeneCard database (https://www.genecards.org/). For the analysis of RNA expression profiles and corresponding clinical data, we utilized resources from the TCGA database for Kidney Renal Clear Cell Carcinoma (KIRC) and Kidney Renal Papillary Cell Carcinoma (KIRP) (https://xena.ucsc.edu/). The E-MTAB-1980 renal cancer dataset, comprising expression profiles and clinical data, was also retrieved from the Array Express database (https://www.ebi.ac.uk/biostudies/arrayexpress). The TCGA-KIRC dataset contained 535 tumour samples and 72 normal tissue samples, while the TCGA-KIRP dataset included 285 tumour samples. Both the KIRC and KIRP contain RNA expression profiles of the 60,000 + gene. For the duplicated genes, we calculated their mean expression levels, and after expelling gene probes without their own corresponding annotation, we got the RNA expression profile of 58,387 genes. The E-MTAB-1980 dataset contained 100 tumour samples and an expression profile of 25,988 genes. RNA expression values from the TCGA-KIRC dataset were standardized and converted to log2(TPM + 1) format. The E-MTAB-1980 and TCGA-KIRP datasets were reserved for subsequent model validation. Furthermore, we sourced immune therapy datasets, including the Alexandra cohort [16] and the GSE78220 cohort from GEO (https://www.ncbi.nlm.nih.gov/gds), to predict responses to immune therapy. Both the Alexandra cohort and GSE78220 contain 25 tumour patients with therapeutic information. Alexandra cohort includes an RNA expression profile of 33,077 genes, and the GSE78220 includes an expression profile of 25,268 genes. We expelled the samples for all datasets without their own prognostic data (including survival status and follow-up times).

To validate the gene expression alteration, four datasets were obtained from the GEO database, including GSE53757, GSE15641, GSE40435, and GSE6344. GSE53757 contains 72 tumour tissues and 72 normal tissues. GSE15641 contains 57 tumour tissues and 23 normal tissues. GSE40435 contains 101 tumour tissues and 101 normal tissues. GSE6344 contains 10 tumour tissues and 10 normal tissues. All expression data were normalized and transferred into log(FPKM + 1) format.

### Screening of core genes related to manganese metabolism

To identify differentially expressed genes (DEGs), we employed the limma package (https://www.bioconductor.org/) to contrast tumour and normal samples within the TCGA-KIRC dataset. Genes with an absolute log fold change (|LogFC|) greater than 0.75 and an adjusted p-value less than 0.05 were classified as DEGs. The intersection of the differentially expressed genes in KIRC and manganese metabolism-related genes was taken to identify manganese metabolism-related differential expressed genes. These genes were then integrated with clinical data from TCGA-KIRC, and univariate Cox regression analysis was conducted using the survival package. The log-rank test was employed to evaluate prognostic differences, with manganese metabolism-related genes exhibiting a p-value less than 0.01 being designated as prognostic genes in KIRC. A hazard ratio (HR) exceeding 1 indicated a risk gene, whereas an HR below 1 indicated a protective gene. Subsequently, the expression data of these prognostic genes, along with patient survival data, were subjected to three machine learning algorithms: Gradient Boosting Machines (GBM), Boruta, and Random Survival Forests (RSF). Previous research chose multiple machine learning algorithms to screen variables [17]. In many research, Boruta measures the importance of features by comparing them with those of shadow variables, and it is robust for both high-dimensional and low-dimensional data [18, 19]. Random Survival Forests (RSF) are data-driven learning algorithms to analyze right-censored survival data. It is fully nonparametric, requires no restrictive assumption, and can automatically deal with the nonlinear effects and high-level interactions among the variables [20]. GBM is a flexible ensemble learning technique that combines gradient-based optimization and boosting techniques, which also make automatic feature selection, prioritizing important variables and discarding ones containing irrelevant or redundant information [21]. Therefore, we choose these three algorithms to conduct variable selection.

For the Boruta, we provided the survival time, outcome, and gene expression to the “boruta” R function from the “Boruta” R package; the parameter “doTrace” was set to 2. The genes that were identified as "confirmed" and “Tentative” were selected, and the genes that were regarded as "rejected" were expelled.

For RFS, we used the “rfscc” R function from the “randomForestSRC” R package; we submitted the survival time, outcome, and gene expression to “rfscc”, the parameter “importance” was True, “ntree” was 100, “nsplit” was 10, “block.size” was 1, then we used the “var.select” R function to screen variables, the parameter “conservative” was “low”.

For GBM, we used the “gbm” R function from the “GBM” R package; survival time, status, and gene expression were provided to “gbm”, the parameter distribution was “coxph”, “n.trees” was 10,000, “intersection.depth” was 3, “n.minobsinnode” was 10, “shrinkage” was 0.001, “cv.folds” was 10, and “n.cores” was 6. Then, we used the function “gbm.perf” to screen variables based on the “OOB” method parameter; the gene with rel.inf > 2 was selected.

These three machine learning methods were utilized to screen genes further, ultimately identifying 11 manganese metabolism core genes (MMCGs).

### Characterization of MMCGs

The expression data for the 11 MMCGs in both tumour and normal tissues from the TCGA-KIRC dataset were extracted and visualized using box plots to delineate gene expression levels between tumour and normal tissues. Protein expression patterns of the MMCGs were examined via the Human Protein Atlas (HPA) immunohistochemistry database (https://www.proteinatlas.org/). Gene dependency scores (gDS) were retrieved from public databases [22], with lower scores indicating the gene's essentiality for tumour cell survival. A gDS score below– 1 signifies that the gene's loss is lethal to tumour cells, a score between − 1 and 0 suggests that the gene's loss affects cell viability, and a score above 0 implies no impact on cell viability. This gDS score system allows us to gauge the significance of genes within tumour cells preliminarily. For pathway analysis, cancer-related Hallmark pathways from the MSigDB database and gene ontology (GO) gene sets (cellular component, molecular function, and biological process) were downloaded. The GSVA package was utilized to calculate the pathway activity score, and then the Pearson method was used to compute the correlation coefficient between pathway activity and expression of 11 MMCGs. The top ten pathways and gene ontology terms associated with each gene were extracted and visualized in network diagrams.

To explore the protein–protein interaction (PPI) of MMCGs, we submitted these 11 MMCGs to the GeneMANIA website (https://www.string-db.org/) to visualize the interaction between MMCGs. To explore the co-expression relationship, we calculated the correlation coefficient between the RNA expression of these 11 genes based on the Pearson method based on the “Hmisc” R package, the result was visualized by “corrplot” R function.

### Construction of MMCG Subtypes

We extracted RNA expression data of the 11 manganese metabolism-related core genes (MMCGs) from the TCGA-KIRC dataset and conducted unsupervised hierarchical clustering using the “factoextra” R package. The Euclidean distance was calculated to measure the dissimilarity between samples, and the Ward.D2 method was applied for clustering. This analysis stratified the TCGA-KIRC dataset into two distinct clusters—named Cluster I and Cluster II—facilitating the visualization of MMCG expression variances through heatmaps.

### Establishment and validation of the prognostic model for MMCGs

We integrated the MMCG expression data with TCGA-KIRC clinical data and then constructed a risk model. Based on ten-fold cross-validation, the Lasso-Cox method was used to select the optimal regularization parameter (λ value). By minimizing the sum of squared residuals while constraining the sum of the absolute values of the parameters, this method generates sparse solutions, causing the coefficients of some unimportant variables to be zero. This approach can avoid overfitting caused by overly complex models. We finally generated a risk score based on the coefficients and gene expression values of the MMCGs based on the “glmnet” R package. The risk score equation is delineated as follows:

$$ {\text{Riskscore }} = {\text{ Coef}}_{{{\text{gene1}}}} *{\text{Expression}}_{{{\text{gene1}}}} +... + {\text{Coef}}_{{{\text{genen}}}} *{\text{Expression}}_{{{\text{genen}}}} $$

The optimal cut-off value for the risk score was determined using the “surv_cutpoint” R function from the “survminer” R package, which segregated the TCGA-KIRC data into high-risk and low-risk cohorts. The prognostic accuracy of the risk model was evaluated via Kaplan–Meier survival curves and Receiver Operating Characteristic (ROC) curve analyses, employing the “survivalROC” R package to quantify the area under the ROC curve (AUC). These analyses were replicated in the E-MTAB-1980 and TCGA-KIRP datasets to ascertain the universality of the risk score.

To validate the risk model as an independent prognostic factor, we integrated risk score with patient clinical variables and conducted multivariate Cox regression analysis, identifying significant variables as independent prognostic factors. To further ascertain the robustness of our risk prediction model, we classified the patients into different clinical subgroups (male and female), and then the Kaplain-Meier survival curves were used to show the prognostic differences between high-risk and low-risk patients.

To compare the risk model accuracy, we used the other algorithms (including Cox regression, ridge regression, and plsRcox regression analysis) to construct the risk model. For the Cox regression, we used the multiple Cox regression analysis based on the stepwise methods. For ridge regression, we used the “glmnet” R function based on tenfold cross-validation, the parameter “alpha” was set as 0. For the plsRcox, we submit the survival data and gene expression data to “plsRcox” function; the parameter nt was set as 5, and alpha.pvals.expli was set to 0.01. These three methods could generate gene coefficients, then we calculate the risk score based on the function in the same way as lasso-cox regression. The time-dependent ROC curve was used to compare the accuracy of the risk model.

### Establishment of the nomogram model

Independent prognostic factors identified from the multivariate Cox regression were utilized to construct a clinical nomogram model. We leveraged the “rms” R package to develop a nomogram model for prognosis prediction, offering a more precise prognostic estimate for clinical patients based on risk scores and clinical characteristics. Discrimination accuracy was estimated using areas under the curve (AUC) and calibration curves. AUC values exceeding 0.7 were considered reasonable estimates. Decision curve analysis (DCA) was employed to assess the clinical utility of the nomogram model.

### Tumor immune infiltration and drug sensitivity prediction

The proportion of 22 immune cell types in each sample was quantified using cell type identification by estimating relative subsets of RNA transcripts (CIBERSORT). Gene sets representing 24 immune cell types were obtained from published articles, and the immune infiltration levels of these cells were calculated using single-sample gene set enrichment analysis (ssGSEA) from the GSVA R package [23]. Based on publicly available websites, we explored the TIDE scores of different patients to assess patients' responses to immunotherapy using a public website (http://tide.dfci.harvard.edu). For drug sensitivity analysis, the IC50 values for specific targeted drugs were calculated for each sample using the “pRRophetic” R package, exploring the sensitivity of different risk group patients to these drugs.

### Mechanistic analysis of subtypes and risk scores

For the mechanism analysis, we first conducted differential expression analysis between Cluster I and Cluster II subtypes, as well as high-risk and low-risk groups. Then, the DEGs were submitted to the DAVID website for enrichment analysis. Furthermore, we ranked the TCGA-KIRC sample based on risk score, and then the whole gene expression profile was submitted to the GSEA software for enrichment analysis. Then, the barplot was visualized to show the enrichment results.

### Statistical analysis

Unpaired t-tests were employed to compare the differences between the two groups. The Pearson correlation coefficients from the Pearson method were used to assess the correlation between two variables. The Chi-square test was carried out to determine differences in the distribution of contingency table data. Univariate and multivariate Cox regression analyses were conducted to evaluate the relationship between variables and patient prognosis, and the log-rank test was used to assess prognostic differences. In all analyses, a p-value less than 0.05 was considered statistically significant, denoted as follows: P < 0.05, *; P < 0.01, **; P < 0.001, ***; P < 0.0001, ****.

## Results

### Screening of manganese metabolism core genes (MMCGs)

**Fig. 1:**
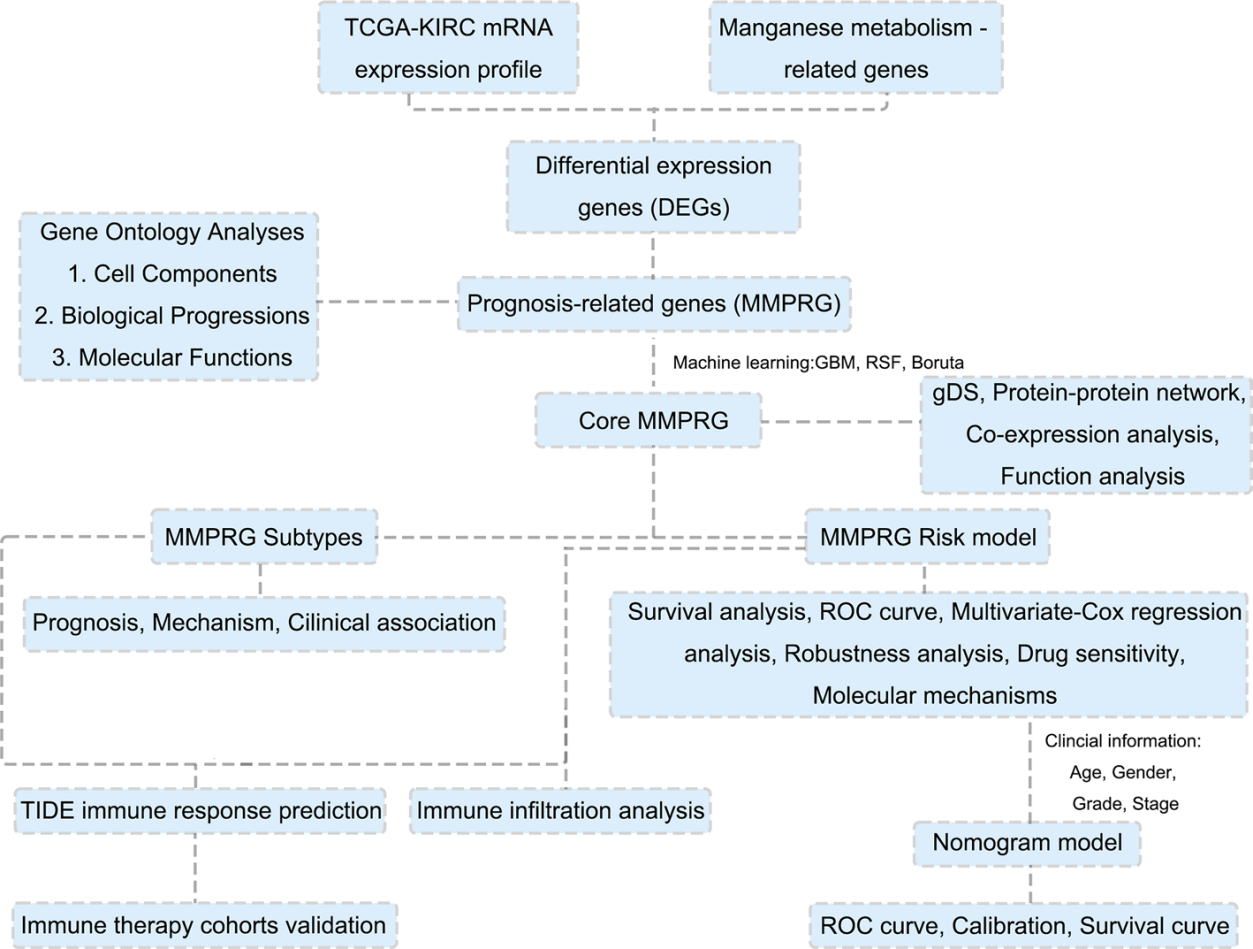
The Flow Chart of this study

**Fig. 2:**
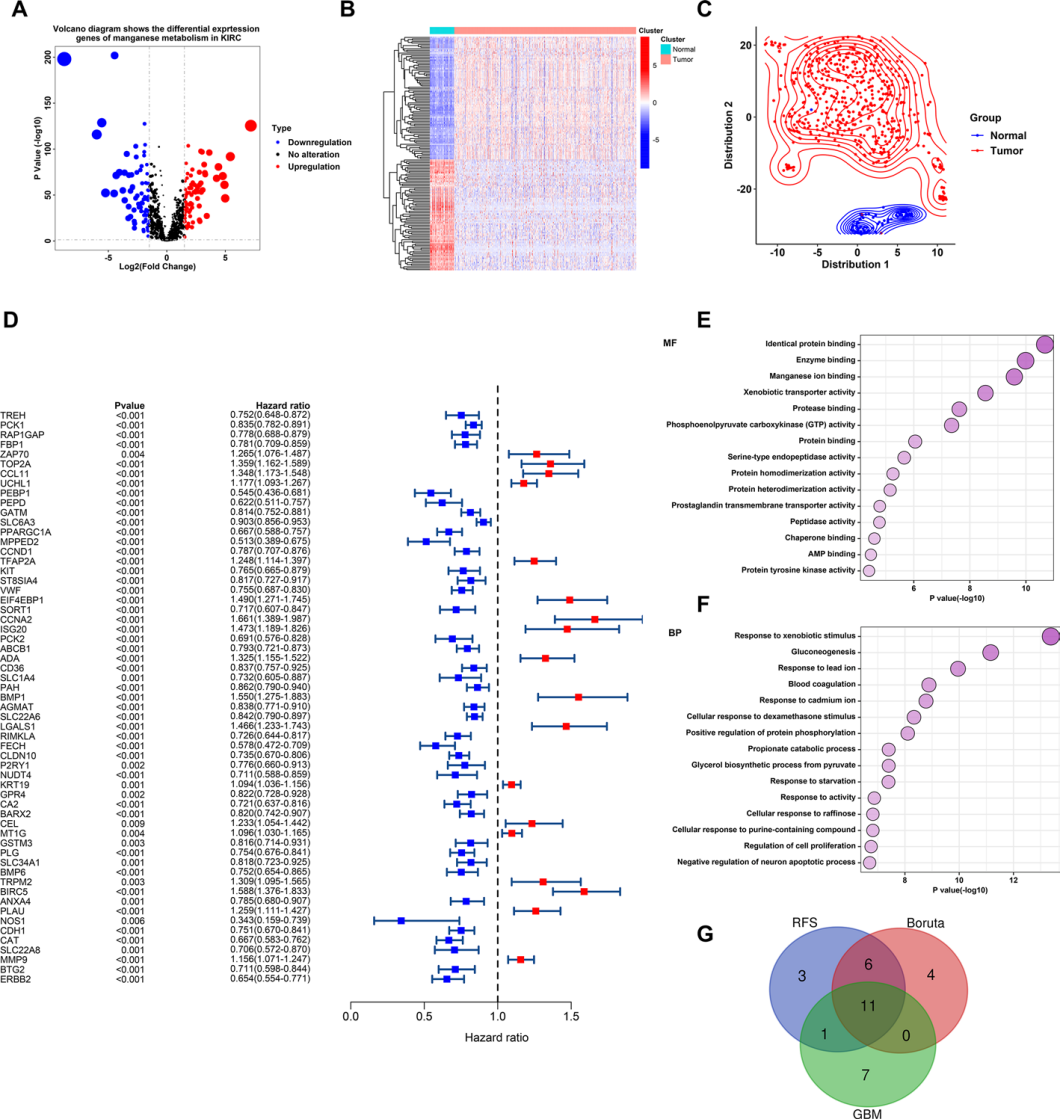
Identification of Manganese Metabolism-Related Core Genes (MMCGs) **A** The volcano plot shows the differentially expressed manganese metabolism-related genes. **B** The heatmap represents expression levels of differentially expressed manganese metabolism-related genes across tumour and normal tissues. **C** T-SNE plot shows the distribution of tumour and normal samples on a two-dimensional coordinate axis based on manganese metabolism-related gene expression. **D** The forest plot shows the prognosis-related manganese metabolism genes; red indicates risk genes, and blue indicates protective genes. **E–F** Bubble plots highlight the enrichment of prognosis-related manganese metabolism genes in molecular functions (MF) and biological processes (BP). **G** Venn diagram shows the manganese metabolism core genes through three distinct machine learning algorithms

The flowchart in this study is shown in Fig. 1. Initially, we extracted manganese metabolism-related genes from the TCGA-KIRC dataset and employed the “limma” R package to calculate differential gene expression (DEGs) (Fig. 2A). The heatmap demonstrated that these differentially expressed manganese metabolism genes could effectively differentiate between tumour and normal samples (Fig. 2B). Furthermore, TSNE analysis revealed that the expression patterns of these genes varied significantly between tumour and normal tissues on the two-dimensional coordinate axes, indicating that the expression levels of these genes are markedly distinct (Fig. 2C). To identify prognosis-related genes, we conducted univariate Cox regression analysis and found that 59 manganese metabolism genes are related to the prognosis of KIRC patients (Fig. 2D). Pathway enrichment analysis revealed that these genes are predominantly related to molecular functions (MF) such as protein binding, enzyme binding, manganese ion binding, xenobiotic transporter activity, and protease binding, as well as biological processes (BP), including response to xenobiotic stimuli, gluconeogenesis, response to lead ions, blood coagulation, and response to cadmium ions (Fig. 2E, F). Considering 59 prognosis-related genes could cause unstable feature selection for further risk model construction, we further screen the core manganese metabolism-related genes by utilizing three machine learning algorithms (GBM, Boruta, and RSF) (Details in Table S1). The Venn diagram revealed that these three machine-learning approaches collectively identified 11 manganese metabolism genes. We named these genes as Manganese Metabolism Core Genes (MMCG) and used them to conduct further analysis (Fig. 2G).

### Characterization of manganese metabolism core genes

We further characterized the 11 Manganese Metabolism Core Genes (MMCGs) and discovered that EIF4EBP1, RIMKLA, and BIRC5 exhibit higher expression in tumour tissues, whereas CEL, MPPED2, PCK1, CAT, PLG, PPARGC1A, PEBP1, and TFAP2A show lower expression in tumour tissues (Fig. S1A). Immunohistochemical database analysis reveals that EIF4EBP1, RIMKLA, and BIRC5 are highly expressed in tumour tissues, while the PCK1, PLG, PEBP1, and CAT show a lower expression in tumour samples. However, data for the remaining genes was unavailable in the HPA database (Fig. S1B). To further validate the expression of MMCG, we utilized four RCC datasets (including GSE6334, GSE40435, GSE15641, and GSE53757) downloaded from the GEO database (Fig. S2A–D). The results revealed that EIF4EBP1, RIMKLA, and BIRC5 upregulated in tumor tissues, while other genes downregulated in normal tissues, which is consistent with the results in TCGA-KIRC datasets. To assess the importance of these 11 MMCGs, we employed Gene Dependency Scores (gDS) and found that BIRC5, PLG, and EIF4EBP1 had gDS scores below zero in most renal cancer cell lines (Fig. S3A). BIRC5 had the lowest gDS score, suggesting that its absence significantly affects tumour cell viability. We then explored the Gene Ontology (GO) terms and pathways associated with these 11 MMCGs by calculating the correlation coefficient between pathway activity scores and the expression level of MMCGs. The top ten pathways with the highest correlation coefficient for each gene were selected to construct a pathway network diagram (Fig. S3B–E, detailed in Table S2). Our findings revealed that MMCGs are involved in various pathways. For instance, BIRC5 is significantly positively correlated with E2F Targets, G2M checkpoint, MYC Target V1, MYC Targets V2, and MTORC1 signalling, while significantly negatively correlated with Adipogenesis, Fatty acid metabolism, oxidative phosphorylation, bile acid metabolism, and heme metabolism. In the GO analysis, BIRC5 is significantly positively correlated with biological processes such as POSITIVE REGULATION OF MITOTIC CELL CYCLE SPINDLE ASSEMBLY CHECKPOINT, REGULATION OF MITOTIC NUCLEAR DIVISION, NEGATIVE REGULATION OF CHROMOSOME SEGREGATION, CHROMOSOME SEPARATION, and POSITIVE REGULATION OF CHROMOSOME SEPARATION. In summary, these data provide a comprehensive characterization and function of MMCGs. Through single-cell data analysis, we obtained precise localization for the 11 MMCGs. In two representative KIRC datasets (GSE159115 and GSE171306), we observed that genes like PLG, PCK1, and CAT are primarily expressed in epithelial cells, PEBP1 is mainly expressed in malignant cells, mononuclear macrophages, endothelial cells, and even CD8 + T cells. TFAP2A, PPARGC1A, MPPED2, and CEL are expressed at lower levels and found only in small epithelial cells. BIRC5 shows a strong expression in Tprolif (Fig. S4A and B). To further elucidate the interrelationships among the 11 MMCGs, we constructed a protein network using the geneMANIA website, revealing complex protein–protein interactions among the MMCGs. Additionally, this website analyzed potential interacting target proteins for these 11 genes (Fig. S5A). Furthermore, we identified significant co-expression relationships among the 11 genes. For instance, PEBP1 is significantly positively correlated with PCK1, PLG, CAT, and PPARGC1A, while it is significantly negatively correlated with CEL, BIRC5, and TFAP2A (Fig. S5B).

### Manganese metabolism subtype construction and mechanism exploration

**Fig. 3:**
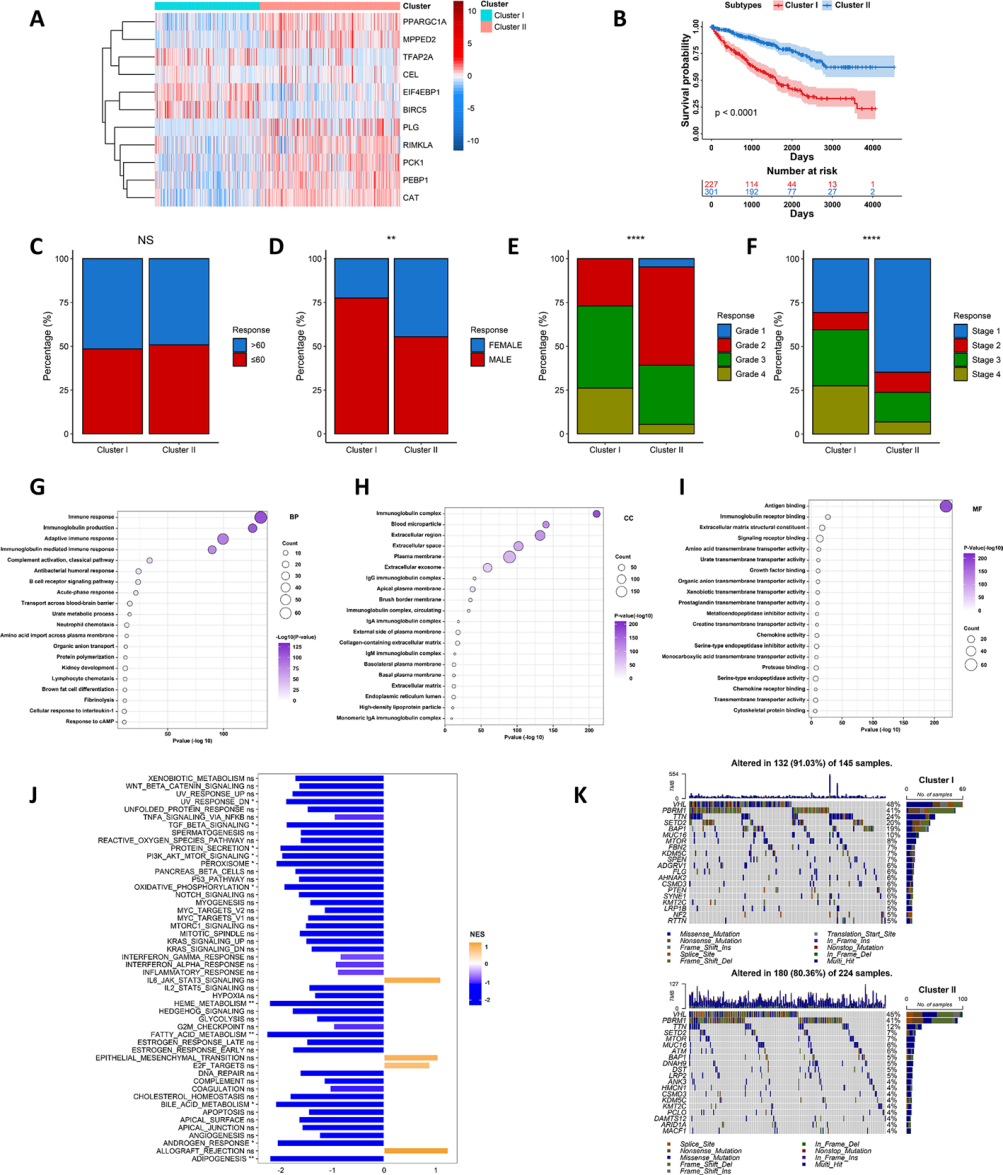
Clinical relevance of manganese metabolism subtypes in the KIRC dataset** A** Heatmap shows expression levels of MMCGs in Manganese Metabolism Clusters I and II. **B** The survival curve shows the clinical outcomes of the patients in Manganese Metabolism Clusters I and II. **C**–**F** Correlations between manganese metabolism subtypes and clinical information (age, gender, grade, and stage). **G**–**I** Bubble plots show the gene ontology (BP, biological process; MF, molecular function; CC, cell component) enrichment of differentially expressed genes between Manganese Metabolism Clusters I and II. **J** Barplot depicted GSEA hallmark pathway enrichment between Cluster I and Cluster II. **K** Mutation characterization of KIRC patients across MMCG Clusters I and II

Based on the expression profiles of the 11 MMCGs, we computed the Euclidean distance among samples to stratify patients into two different subtypes—Cluster I and Cluster II. In Cluster I, genes such as TFAP2A, CEL, EIF4EBP1, and BIRC5 were found to be upregulated, whereas the remaining genes were upregulated in Cluster II (Fig. 3A). Survival analysis revealed that patients in Cluster I had a poorer prognosis than those in Cluster II (Fig. 3B). Notably, patient age was not associated with manganese metabolism subtypes. A higher proportion of male patients was observed in Cluster I, and there was a greater prevalence of patients with high histological grades (Grade III and Grade IV) and advanced stages (Stage III/Stage IV) in this cluster (Fig. 3C–F). These findings suggest that Cluster I is associated with advanced stage and grade. We further identified differentially expressed genes (DEGs) between Cluster I and II patients and performed functional enrichment analysis. The results indicated that these DEGs were enriched in different biological processes (BP), cellular components (CC), and molecular functions (MF) (Fig. 3G–I). Additionally, we found that several pathways lowly enriched in Cluster I, including TGF-BETA signalling, protein secretion, PI3K AKT MTOR signalling, peroxisome, oxidative phosphorylation, fatty acid metabolism, bile acid metabolism, and androgen response (Fig. 3J). Mutation feature analysis revealed that Cluster I shows a higher mutation burden (Fig. 3K). We conducted the same analysis in two additional renal cancer datasets (E-MTAB-1980 and KIRP), and the results consistently demonstrated that the prognosis of patients in Cluster I was significantly poorer than those in Cluster II. We also observed a higher proportion of patients with advanced stages and grades in Cluster I (Fig. S6A–K). In conclusion, these results suggest that the MMCG subtypes can serve as predictive markers for disease progression and patient prognosis in Renal Cell Carcinoma (RCC).

### Construction of MMCGs risk model

**Fig. 4:**
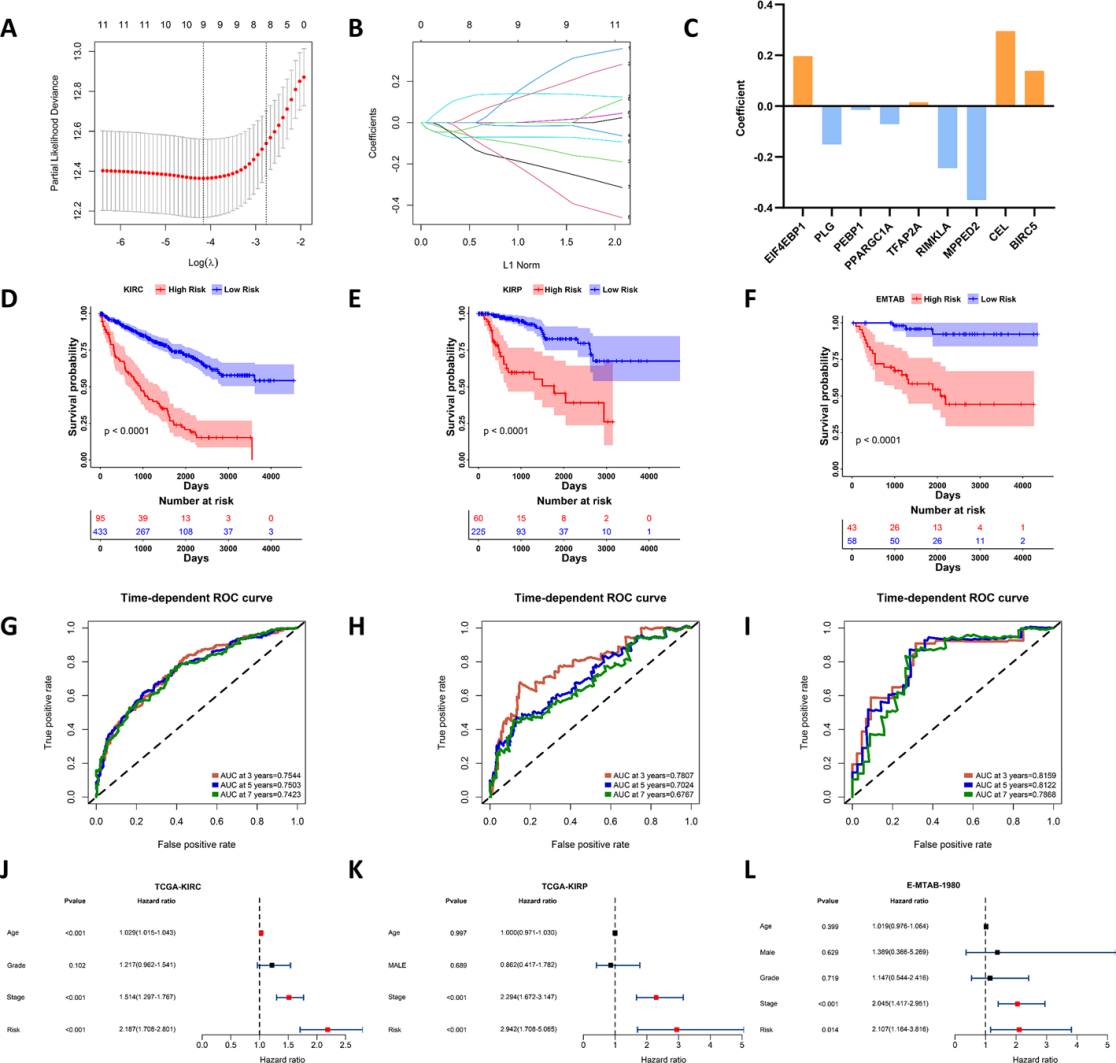
Construction of MMCGs Risk Model** A**, **B **Lasso-Cox regression analysis process. **C** The bar plot represents the lasso regression coefficients of MMCG genes. **D–F** Survival curves demonstrate the differences in clinical outcomes between high and low MMCG risk groups across three independent RCC datasets. **G**–**I** ROC curves assess the predictive accuracy of the MMCG risk score for patient survival at 3, 5, and 7 years. **J**–**L** Multivariate Cox regression analysis, adjusting for age, gender, stage, grade, and MMCGs to determine the independent prognostic factors

To construct the MMCG risk model, we conducted LASSO-cox regression analysis on the KIRC dataset (Fig. 4A and B). 9 genes were identified to construct the risk model **(**Fig. 4C**)**. The formula for calculating the risk score is:

$$ \begin{array}{l} {\text{Riskscore}} \quad= 0.196909188 \times {\text{EIF4EBP1}} - 0.150973197 \\ \hspace{3cm} \times {\text{PLG}} - 0.014800812 \times {\text{PEBP1}} - 0.070778384 \\ \hspace{3cm} \times {\text{PPARGC1A}} + 0.015132806 \times {\text{TFAP2A}} - 0.243351546 \\ \hspace{3cm} \times {\text{RIMKLA}} - 0.369504739 \times {\text{MPPED2}} + 0.295414507 \\ \hspace{3cm} \times {\text{CEL}} + 0.1387384 \times {\text{BIRC5}} \\ \end{array} $$

Utilizing the “surv_cutpoint” function from the “survminer” package, we determined the optimal cut-off value for dividing patients into high and low MMCG risk groups. We found that patients classified as high MMCG risk had significantly poorer outcomes than those in the low MMCG risk group in the KIRC dataset (Fig. 4D). The ROC curve shows that the predictive accuracy of the risk score was high, with AUC values at 3, 5, and 7 years being 0.7544, 0.7503, and 0.7423, respectively (Fig. 4G). Consistent results were observed in two additional RCC datasets: KIRP with AUC values of 0.7807, 0.7024, and 0.6767 at 3, 5, and 7 years, respectively, and E-MTAB-1980 with AUC values of 0.8159, 0.8122, and 0.7866 at 3, 5, and 7 years (Fig. 4E, F, H, I). Furthermore, we conducted a multivariate Cox regression analysis by integrating the risk score with clinical variables. The results showed that the risk score is an independent prognostic factor across three independent RCC datasets (Fig. 4J–L). To evaluate the robustness, we stratified patients into different clinical subgroups based on various clinical variables (for example, based on age, the patients were classified into age greater than 60 and less than 60 years old groups). Then, we divided the patients of subgroups into high and low-MMCG-risk groups and further used the Kaplain-Meier survival curve to analyze the survival rate of different groups. Consistently, whatever subgroups, high MMCG-risk patients always show a worse prognosis than low MMCG-risk patients, indicating that our risk score is robust to predict prognosis in different subgroups (Fig. S7A–C). We then collected some existing risk models that have been published to conduct a comparison with our MMCG risk model, including CRFGs [13, 24], DEGRGs [25], HM [26], LRPS [27], and MMG [28]. ROC curves were used to compare the predictive accuracy. Our model showed a higher accuracy in predicting the 3-/5-/7 year prognosis of KIRC patients than other 5 risk models, indicating that our model has a high superiority in prediction (Fig. S8A-C). In addition, we used three algorithms to construct risk scores (details in Table S3). ROC curve shows that the predicting accuracy of the ridge regression model (AUC at 3 years = 0.7544, AUC at 5 years = 0.7504, and AUC at 7 years = 0.7423) was consistent with lasso-cox regression. The predicting accuracy of other two algorithms is lower than lasso-cox regression model (for Cox regression model: AUC at 3 years = 0.7033; AUC at 5 years = 0.706, AUC at 7 years = 0.6755; for plsRcox regression model: AUC at 3 years = 0.7314, AUC at 5 years = 0.7385, and AUC at 7 years = 0.7345) (Fig S8D–F). Considering that lasso-cox regression is widely used for construction of risk model, we selected lasso-cox regression model to further research.

### Relationship between the MMCGs risk model and clinical factors

**Fig. 5:**
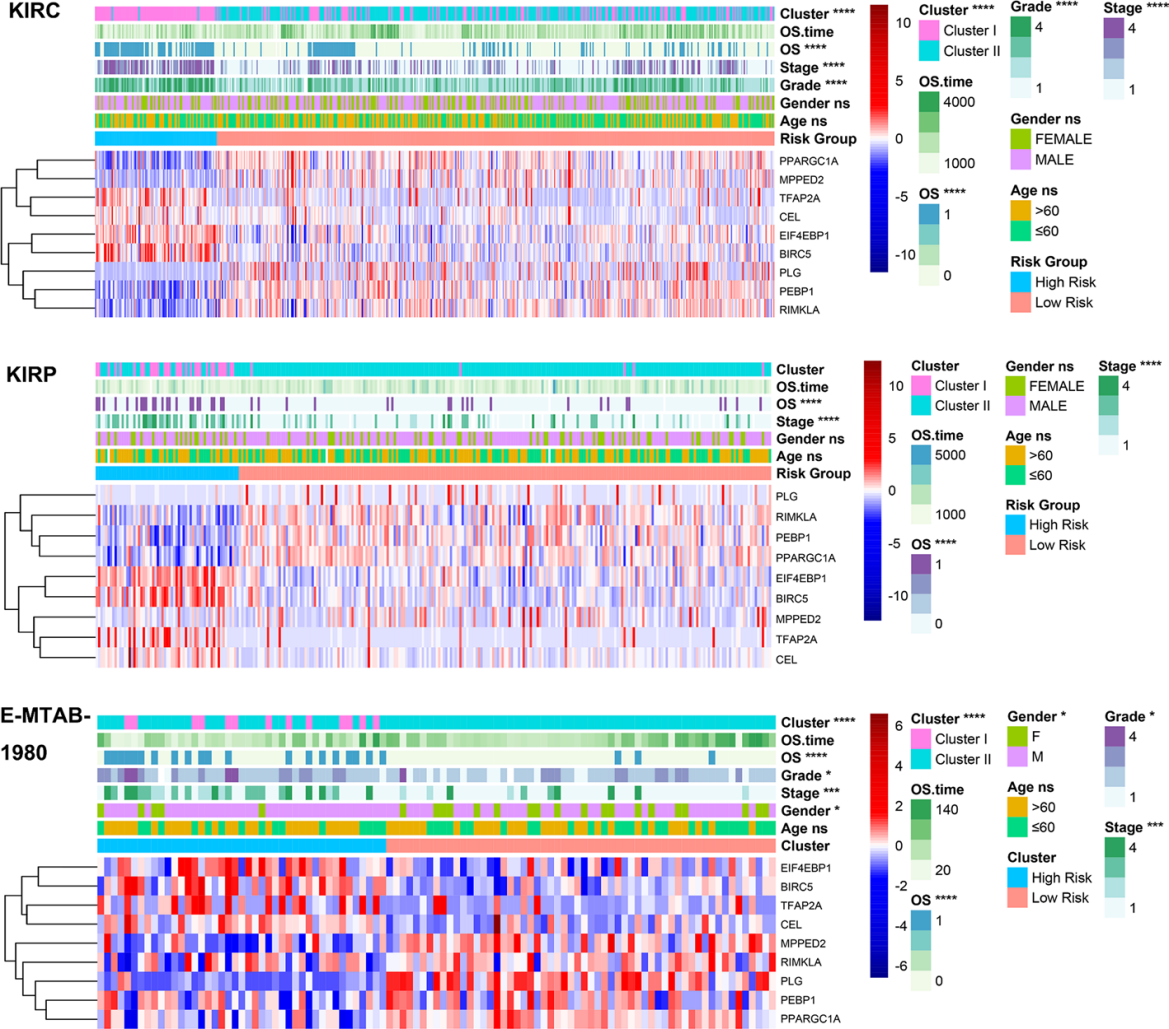
Heatmap shows the distribution differences of MMCG expression, patient age, gender, stage, grade, and MMCG subtypes in high and low MMCG risk groups across three independent RCC datasets

**Fig. 6:**
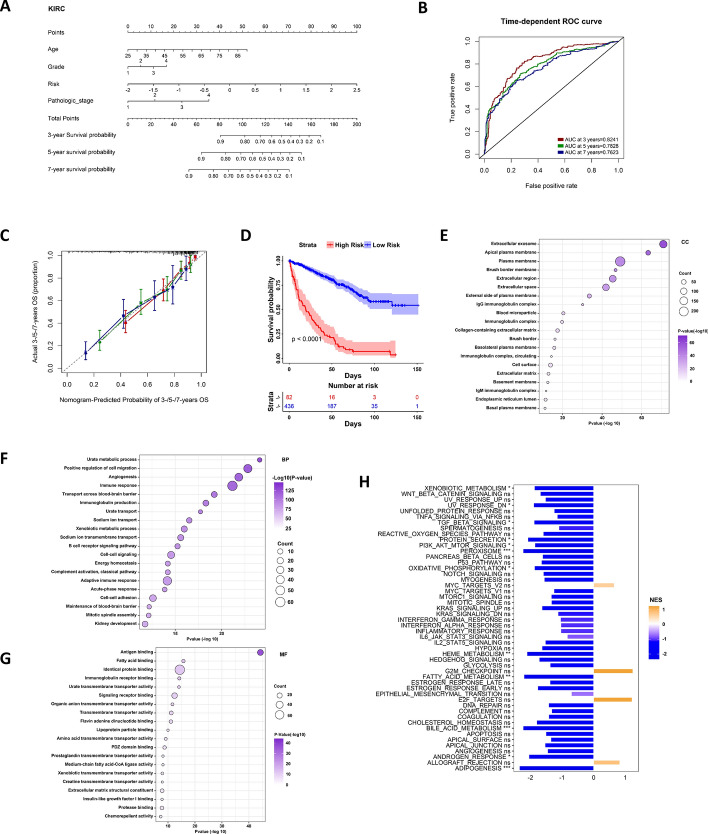
Clinical Relevance, Nomogram Model, and Molecular Mechanisms** A** Nomogram predicts the survival rates of KIRC patients at 3, 5, and 7 years. **B, C** ROC curves and calibration curves evaluate the predictive accuracy of the nomogram in predicting a 3-/5-/7-year survival rate. **D** Survival curves show the prognostic differences between high-risk and low-risk groups based on the nomogram. **E–G** Bubble plots show the gene ontology enrichment (GO, BP, and CC) of differentially expressed genes between high-risk and low-risk groups. **H** Barplot shows GSEA hallmark pathway enrichment between high-risk and low-risk groups

In our analysis across three independent Renal Cell Carcinoma (RCC) datasets, we examined the distribution of various clinical indicators between the high and low Manganese Metabolism Core Genes (MMCGs) risk groups. The findings revealed significant disparities in staging, grading, and survival status between these groups (Fig. 5). Notably, a higher proportion of patients with advanced stage, high grade, and poorer survival status were identified in the high MMCGs risk group. Moreover, patients with poorer prognosis in Cluster 1 were predominantly located in the high MMCG risk group. Genes such as TFAP2A, CEL, EIF4EBP1, and BIRC5 were upregulated in the high MMCGs risk group, while other genes were upregulated in the low MMCGs risk group. We then provided the significant variables identified from the multivariate Cox regression to stepwise R function to select further variables based on the minimum AIC value; the age, grade, stage, and MMCG risk score were determined to construct a nomogram (Fig. 6A). The nomogram was used to estimate the survival probabilities of renal cancer patients at 3, 5, and 7 years. ROC curve revealed a high accuracy of this nomogram, with AUC values of 0.8241, 0.7828, and 0.7623 at 3, 5, and 7 years, respectively (Fig. 6B). Calibration curves further confirmed the high accuracy of the model in predicting survival at 3, 5, and 7 years (Fig. 6C). Based on the nomogram, patients were stratified into high-risk and low-risk groups, and a significant survival difference was observed between these two groups (Fig. 6D). Similar analyses in two additional RCC datasets also demonstrated high predictive capability (Fig. S9A–H). We further conducted enrichment analysis on differentially expressed genes between the high-risk and low-risk groups. The results indicated enrichment in cellular components such as extracellular exosome, apical plasma membrane, plasma membrane, brush border membrane, and extracellular region; molecular functions including urate metabolic process, positive regulation of cell migration, angiogenesis, immune response, and transport across the blood–brain barrier; and molecular functions like antigen binding, fatty acid binding, identical protein binding, immunoglobulin receptor binding, and urate transmembrane transporter activity (Fig. 6E–H). Furthermore, pathways such as XENOBIOTIC METABOLISM, UV_RESPONSE_DN, PROTEIN_SECRETION, PI3K AKT MTOR SIGNALING, OXIDATIVE PHOSPHORYLATION, HEME METABOLISM, FATTY ACID METABOLISM, BILE ACID METABOLISM, ANDROGEN RESPONSE were found to be underrepresented in the high-risk group.

### Immune infiltration and drug treatment

**Fig. 7:**
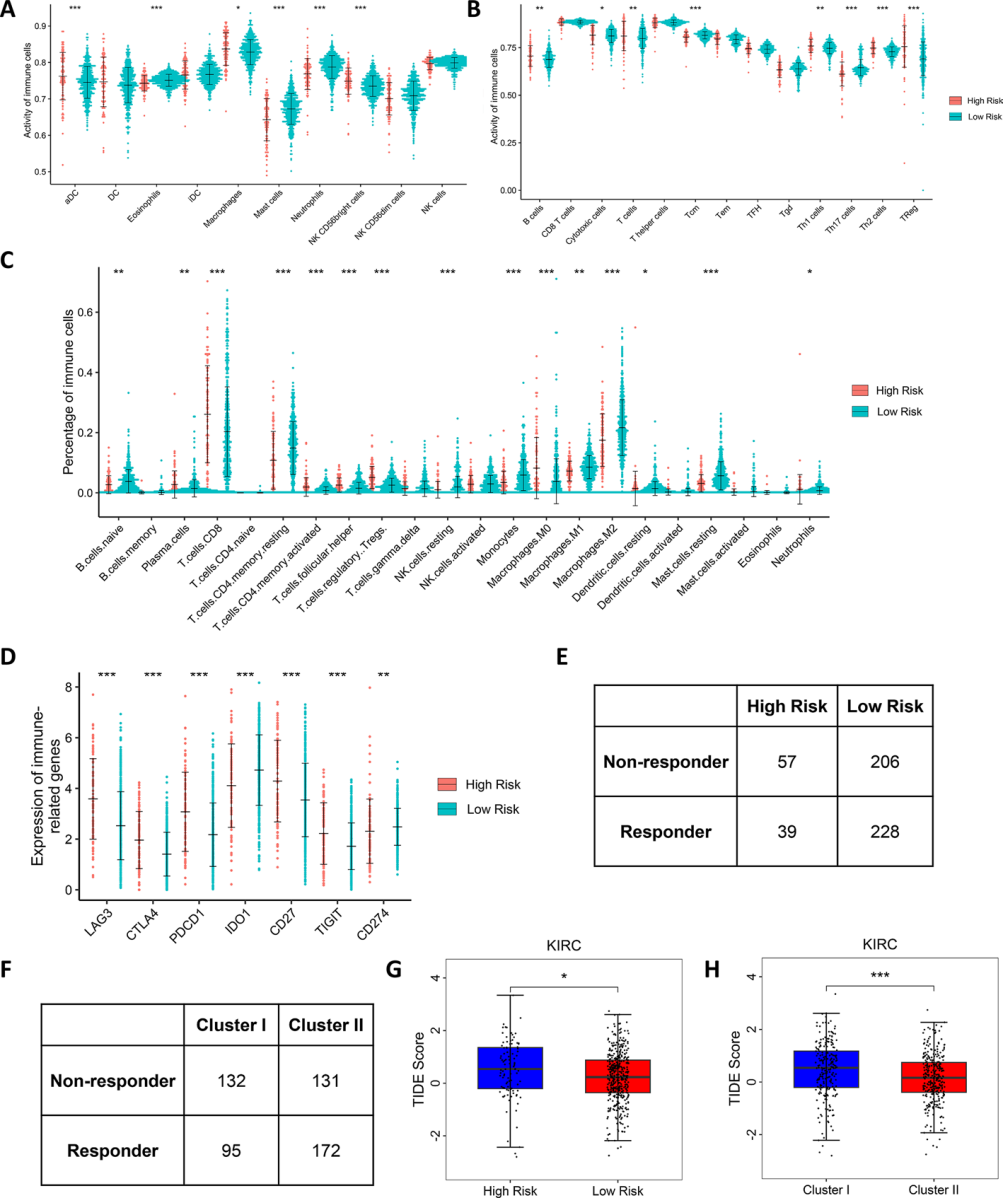
Immune Infiltration and Immunotherapy Response across MMCG Risk Groups. **A**, **B** Immune infiltration levels of innate and adaptive immune cells between MMCG risk groups. **C** Differences in the proportions of 23 immune cell types within MMCG risk groups. **D** Expression differences of seven immune checkpoints between high-risk and low-risk patients. **E**, **F** Tables compare the distribution of immune-responsive and non-responsive patients between high-/low-risk groups and Clusters I/II. **G**, **H** Boxplot compares the TIDE scores in high-risk and low-risk groups and Cluster I/II patients

Given that the enrichment analysis results suggest a link between MMCG subtypes and risk scores and the immune system, we conducted a deeper investigation into the immune cell dynamics between different risk groups. Our findings indicated an increased activity among innate immune cells such as ADCs (Antibody-Dependent Cellular Cytotoxicity), macrophages, and NK CD56bright cells in the high MMCGs risk group (Fig. 7A). The adaptive immune cell population, as well as anti-tumor immune cells like B cells, cytotoxic cells, T cells, Th2 cells, and Th1 cells, also exhibited increased activity in the high MMCGs risk group. However, heightened activity was observed in pro-tumor immune cells such as Tregs (T regulatory cells) in the same group (Fig. 7B). The proportions of naive B cells, CD8 T cells, and Tregs were significantly higher in the high MMCGs risk group, while the proportions of M1 and M2 macrophages were lower (Fig. 7C). In addition, we calculate the immune infiltration using MCP immune cell annotation; we found CD8 T cells, T cells, and fibroblast show higher activity in the high-risk group, while NK cells show lower activity (Fig. S11A). In terms of immune checkpoint expression, LAG3, CTLA4, PDCD1, CD27, and TIGIT were found to be expressed at higher levels in the high MMCGs risk group, whereas IDO1 and CD274 were expressed at lower levels (Fig. 7D). Although the high MMCG risk group showed signs of immune activation, pro-tumor immune cells like Tregs and fibroblasts indicate a complex immune landscape, complicating the inference of immunotherapy response in high versus low MMCG risk patients. To further elucidate this, we performed a TIDE (Tumor Immune Dysfunction and Exclusion) analysis, which revealed a significantly higher number of patients with no response to immunotherapy in the high MMCG risk group (Fig. 7E). Additionally, there was a notable increase in immune response count in Cluster I (Fig. 7F). The TIDE score was significantly elevated in both the high-risk group and Cluster I, suggesting that patients in these groups may be relatively less sensitive to immunotherapy (Fig. 7G and H). Moreover, drug sensitivity analysis showed that sunitinib and temsirolimus had higher IC50 values in high MMCG risk patients, implying a potential lack of response to these treatments. Conversely, pazopanib and sorafenib had lower IC50 values in the high MMCGs risk group, indicating a possible increased sensitivity to these drugs (Fig. S10A and B). We also calculated the MMCG risk score in two additional immunotherapy datasets, observing the same trend where high MMCG risk patients had a worse prognosis compared to low MMCG risk patients (Fig. S11B and E). ROC curves indicated the predictive accuracy of the MMCG risk score for patient prognosis at 1 to 3 years (Fig. S11C and F). Grouped bar charts showed a significantly higher proportion of SD/PD (Stable Disease/Progressive Disease) patients in the high-risk group (Fig. S11D and G). These results may suggest the potential therapeutic predictive effects of the MMCG subtypes and risk score, offering insights into patient stratification for immunotherapeutic strategies.

## Discussion

In recent years, the global incidence of renal cell carcinoma (RCC) has been on the rise [29]. Despite medical advancements introducing new diagnostic tools and therapeutic strategies that have improved outcomes for early-stage RCC patients, the overall survival rate remains a matter of concern [30, 31]. Therefore, there is an urgent need to discover new biomarkers for RCC prognosis, especially for clear cell RCC (ccRCC), and to chart new courses for targeted therapies.

Manganese (Mn), an essential trace element, is critical in numerous biological processes, including antioxidant defense [32], energy metabolism [33], and cell signalling [34]. By modulating the activity of key enzymes such as manganese superoxide dismutase (MnSOD), glutathione peroxidase, and pyruvate carboxylase, Mn contributes to maintaining normal cellular function and metabolic equilibrium [35].

Recent research indicates that manganese metabolism has a dual role in the tumour microenvironment. On one hand, Mn, as a cofactor for MnSOD, aids in the elimination of reactive oxygen species (ROS) and shields cells from oxidative stress, a precursor to tumorigenesis [36–38]. On the other hand, certain tumour cells alter Mn metabolism to foster rapid proliferation and malignancy by upregulating MnSOD [39]. Moreover, imbalanced manganese intake has been linked to an increased risk of specific cancers, implying a role for manganese metabolism in oncogenesis [40, 41]. However, the relationship between manganese metabolism and tumours, particularly renal cell carcinoma (ccRCC), is still in the preliminary research stage, and its potential importance cannot be overlooked. Thus, we employed bioinformatics to investigate the link between manganese metabolism-related genes and the prognosis and treatment response of ccRCC patients, with the goal of uncovering new biomarker evidence for the precision treatment of ccRCC.

Employing differential expression analysis, univariate Cox regression analysis, and machine learning algorithms, we identified 11 manganese metabolism core genes (MMCGs). Single-cell data analysis localized the expression of these 11 MMCGs, revealing their expression in various cell types, including epithelial cells, malignant cells, mononuclear macrophages, endothelial cells, and CD8 + T cells. Subtype analysis based on these 11 MMCGs showed that patients in cluster 1 had a poorer prognosis than those in cluster II. Gene Set Enrichment Analysis (GSEA) of these subtypes indicated that pathways such as TGF-BETA signalling, PI3K AKT MTOR signalling, peroxisome function, oxidative phosphorylation, fatty acid metabolism, and bile acid metabolism were downregulated in cluster I, all of which are associated with tumour progression [42–47].

The TGF-β pathway serves as a critical signalling pathway in tumours. In early-stage RCC, TGF-β suppresses cell proliferation through Smad-dependent signalling. However, in advanced stages, TGF-β signalling promotes epithelial-mesenchymal transition (EMT), invasion, and metastasis via non-canonical pathways such as PI3K/AKT and MAPK [48]. TGF-β may regulate the expression of manganese transporters (including ZIP8 and ZIP14) through both Smad-dependent and Smad-independent pathways, thereby increasing intracellular manganese concentrations. This elevation enhances MnSOD activity, reduces ROS levels, and promotes tumour cell survival. Manganese may synergistically enhance TGF-β's non-canonical signalling by activating MAPK or NF-κB pathways, collectively driving RCC cell migration and invasion [49–51]. Monitoring TGF-β pathway activity (e.g., p-Smad2/3) and manganese metabolism-related proteins (e.g., ZIP8, MnSOD) could potentially predict RCC progression. Similarly, the PI3K/AKT/mTOR signalling pathway—a star molecular pathway—promotes cell proliferation, survival, angiogenesis, and metabolic adaptation (including enhanced glycolysis), making it a key therapeutic target in RCC [52]. Multilevel interactions may exist between PI3K/AKT/mTOR signaling and manganese metabolism in RCC. Elevated intracellular manganese levels could reduce ROS through MnSOD activity, thereby inhibiting ROS-mediated oxidative inactivation of PTEN and consequently enhancing PI3K/AKT signalling. Conversely, manganese deficiency might increase ROS levels while paradoxically activating pro-survival AKT pathways. As a divalent metal cofactor, manganese ions may directly activate PI3K or AKT (analogous to Mg^2^⁺ function) or competitively inhibit other metal ions (e.g., iron) to modulate pathway activity [53–55]. The dysfunction of fatty acid metabolism is associated with the development of kidney cancer. For example, SCD1 (stearoyl-CoA desaturase) has been found to be upregulated in ccRCC and is related to the activation of HIF-2α and P13K/AKT pathways [56]. The exposure level of manganese is correlated with the level of triglycerides, indicating that manganese might influence Lipid synthesis and metabolism [57]. In addition, Mn supplement or intake could reduce abdominal fat accumulation by decreasing fatty acid synthase and malate dehydrogenase activities in the liver, and glycerol in adipose tissue, as well as decrease total cholesterol, LDL-cholesterol and HDL-cholesterol [58]. Moreover, pharmacological inhibitors of fatty acid synthase could suppress the growth and invasiveness of renal cancer cells [59]. These results indicate that manganese could regulate the progression of kidney cancer through fatty acid metabolism. However, the links between manganese and these cancer-related pathways still need further validation.

Subsequently, we conducted a Lasso regression analysis on the KIRC dataset to develop a more accurate model for predicting patient prognosis. Lasso-cox regression identified nine genes to construct a risk model. Among these, PLG, PEBP1, PPARGC1A, RIMKLA, and MPPED2 were identified as protective factors, while EIF4EBP1, TFAP2A, CEL, and BIRC5 were recognized as risk factors. These genes are individually implicated in tumour biology. For instance, the PLG gene encoding plasminogen is associated with a better prognosis in ccRCC patients when highly expressed [60]. The PEBP1 protein's ubiquitination and degradation can activate the ERK signalling pathway, promoting clear cell renal cell carcinoma progression [61]. MPPED2 upregulation inhibits glioblastoma cell proliferation and enhances temozolomide sensitivity, and it also functions as a tumour suppressor in breast cancer [62, 63]. EIF4EBP1 contributes to carcinogenesis in lung adenocarcinoma, hepatocellular carcinoma, and nasopharyngeal carcinoma, and its suppression inhibits ccRCC cell proliferation, migration, and invasion [64–67]. The TFAP2A gene encodes the TFAP2α protein, which is primarily located in the nucleus. TFAP2α binds to the promoter of TGF-β1 and promotes its transcription, thereby enhancing tumour epithelial-mesenchymal transition (EMT). TFAP2A is significantly overexpressed in most cancer types [68]. Additionally, BIRC5, a proliferation marker, is associated with poor prognosis in breast cancer [69]. Needless to say, these genes play different roles in regulating tumour progression. However, whether they regulate kidney cancer by affecting the manganese metabolism still needs further experimental validation. Based on the risk model, we stratified patients into high and low-MMCG risk groups and observed that high-risk KIRC patients had significantly worse prognoses than low-risk patients. The model's robust and stable predictive performance was validated across three independent kidney cancer cohorts. Most patients in MMCG cluster I were found in the high MMCG risk group, and pathways such as HEME metabolism, fatty acid metabolism, and bile acid metabolism were highly enriched in low MMCG risk groups, consistent with MMCG subtype enrichment. This suggests that differential pathway enrichment may underlie the survival disparity between high and low MMCG risk groups. All in all, our risk model and MMCG subtype provide a practical method for predicting patients' clinical outcomes. For example, for kidney cancer patients, we can detect their expression of MMCG and calculate a risk score; we can also classify them into different subtypes to predict their clinical outcomes. For different patients, we can carry out different clinical therapeutic strategies based on their pathway enrichment activity.

In addition, we predicted the IC50 values of several drugs in high and low MMCG risk groups. Sunitinib and Temsirolimus showed low IC50 values in the high-risk group, indicating that these patients might be sensitive to these drugs. In contrast, low-risk patients might be more sensitive to Pazopanib and Sorafenib. Temsirolimus is an mTOR inhibitor that can inhibit tumour growth by regulating the mTOR signalling pathway [70]. Additionally, exposure to doses of Sunitinib higher than those used clinically has been shown to result in a decline in MCL-1 levels and inhibition of mTOR signalling. However, increased MCL-1 levels and activation of the mTOR signaling pathway are correlated with resistance to Sunitinib [71]. In our research, we found that mTOR pathway activity was low in the high-risk group, which might explain the low IC50 values observed in these patients. Previous studies have indicated that mTOR inhibitors can reverse Sunitinib resistance through combination therapy, suggesting that inhibiting the mTOR pathway might overcome Sunitinib resistance [72]. When the mTOR pathway is over-activated, it may enhance the survival of tumour cells through a feedback loop, leading to resistance to Temsirolimus [73]. In a clinical trial involving patients with advanced solid tumours, researchers found that activation of the mTOR pathway, such as mutations in mTORC1 and mTORC2, was associated with a durable response to Pazopanib. This suggests that activation of the mTOR pathway may be a biomarker of Pazopanib sensitivity in some patients [74]. However, the precise mechanisms underlying these observations remain unclear. Therefore, exploring the potential reasons for the differential sensitivity of KIRC patients to these drugs is essential for developing individualized therapies.

The intricate relationship between the immune system and tumours significantly influences tumorigenesis, progression, and therapeutic response [75, 76]. We detected substantial differences in immune profiles between high and low MMCG risk groups, particularly in regulatory T cells (Tregs) and fibroblast. The high MMCG risk group exhibited increased Tregs and fibroblast infiltration. Tregs can suppress effector T cell functions, such as those of CD8 + T cells, by secreting inhibitory cytokines like IL-10 and TGF-β and through direct cell interactions, thereby impairing their ability to recognize and eliminate tumour cells [77–79]. High fibroblast infiltration always causes poor prognosis and immunotherapeutic response in cancer patients, indicating that fibroblast infiltration does sometimes conspire to inhibit CD8 + T-cell activity [17]. Inhibition of mTOR activity can lead to the activation of autophagy, and Tregs with defective autophagy have been shown to exhibit high levels of mTORC1-Myc pathway activity and glycolytic activity [80]. BIRC5 is positively regulated by the AKT/mTOR pathway to inhibit autophagy and apoptosis and promote cell survival [81]. In addition, BIRC5 could promote cancer progression by modulating PD-L1 expression and inducing tumour immune evasion [82]. In addition, Tregs had elevated phosphorylation of 4E-BP1 (EIF4EBP1), which is a primary mTORC1 downstream target [83]. The activity of EIF4EBP1 influences Lipid synthesis pathways, and Treg function depends on lipid metabolism, implying the important role of EIF4EBP1 in promoting the Treg function [83]. Glycolysis is the primary metabolic mode of CAFs due to the increased expression of HIF-1a and monocarboxylate transporter MCT4 [84]; BIRC5 is also correlated with the activity of glycolysis, implying that the regulational mechanism of manganese on CAFs. BIRC5 is also positively correlated with CAF inflation levels, indicating a synergistic function between BIRC5 and CAFs [85]. BIRC5 could up-regulated PD-L1 expression and inhibit the function of CD8 + T cells, and the CAFs could secrete immune inhibitors (such as IL-6) to promote immune evasion [82]. All in all, these varying immune landscapes dictate patients' responses to immunotherapy, with our findings indicating a higher number of non-responders in the high MMCG risk group and cluster I, potentially explaining their poorer prognosis and low immune response. Thus, MMCG subtypes and risk groups could serve as practical stratification tools to identify patients who are sensitive or resistant to immunotherapy or targeted therapy.

## Limitation

While the MMCG risk signature shows promise in predicting prognosis and immune therapy response, several limitations must be acknowledged. These include variations in cancer types, sample sizes, data formats, and gene detection technologies across different cohorts. Additionally, the retrospective nature of clinical data collection and the lack of detailed therapy-related clinical information may introduce discrepancies and affect model stability. Prospective studies are needed to validate the model's effectiveness as an independent predictor of prognosis and therapy response.

Furthermore, the clinical data don't include geographic and ethnic information, which limits the ability to determine whether the MMCG risk signature can be effectively applied across different locations or ethnic groups. Moreover, the lack of information could influence the prediction accuracy of MMCG risk signatures, such as patient comorbidities, previous treatments, and environmental factors. Additionally, further experimental validation of the molecular mechanisms of MMCGs in KIRC is necessary.

## Supplementary Information

Supplementary material 1. Fig. S1. Expression alteration of MMCGs. (A) Boxplot shows the expression levels of MMCGs in tumors versus normal tissues. (B) Protein expression levels of MMCGs in the HPA database.

Supplementary material 2. Fig. S2. Boxplots show the expression alteration of MMCGs in four independent datasets - GSE63757 (A), GSE15641 (B), GSE40435 (C), and GSE6344 (D).

Supplementary material 3. Fig. S3. Importance Assessment and Molecular Functions of MMCGs. (A) Heatmap shows the gene dependency scores (gDS) of MMCGs across various renal cancer cell lines. (B) The network diagram shows the top 10 cancer-related hallmark pathways significantly associated with the expression of 11 MMCGs. (C) The network diagram illustrates the top 10 biological processes highly related to the expression of 11 MMCGs. (D) The network diagram shows the top 10 molecular functions highly related to the expression of 11 MMCG. (E) The network diagram represents the top 10 cellular components that are significantly associated with the expression of 11 MMCG.

Supplementary material 4. Fig. S4. Expression location of MMCGs in Renal Cancer Single-Cell Sequencing Datasets - GSE159115 (A) and GSE171306 (B).

Supplementary material 5. Fig. S5. Cross-Talk Among MMCGs. (A) The network diagram displays the protein-protein interactions of the 11 MMCGs and their potential binding target proteins. (B) Correlation coefficient plot showing co-expression relationships of the 11 MMCGs based on their expression levels.

Supplementary material 6. Fig. S6. Association between MMCGs Subtypes and Clinical Factors in E-MTAB-1980 and KIRP Datasets. (A, G) Heatmap of expression levels of MMCGs in Manganese Metabolism Cluster I and II. (B, H) Survival differences between patients in Manganese Metabolism Cluster I and II. (C-F, I-K) Correlations between manganese metabolism subtypes and clinical factors (age, gender, grade, and stage).

Supplementary material 7. Fig. S7. Robustness Analysis of MMCGs Risk Score. (A-C) In three independent renal cancer datasets, patients were stratified into different clinical subgroups and survival curves were utilized to assess prognostic differences between high-risk and low-risk groups, thereby evaluating the robustness of the MMCGs risk model.

Supplementary material 8. Fig. S8. Model comparision. (A-C) ROC curve shows the predictive accuracy between different risk models on predicting 3-/5-/7-year survival. (D-F) ROC curve shows the predictive accuracy between different risk models based on different algorithms on predicting 3-/5-/7-year survival.

Supplementary material 9. Fig. S9. Construction of Clinical Nomogram in External Datasets. (A) A nomogram was depicted to predict the survival rates of the KIRP patients at 3-/5-/7-years. (B, C) ROC and calibration curves evaluate the predictive accuracy of the nomogram for patient survival at 3, 5, and 7 years. (D) Survival curves show the prognostic differences between high-risk and low-risk groups based on the nomogram model. (E) A nomogram was depicted to predict the survival rates of patients in the E-MTAB-1980 dataset at 3, 5, and 7 years. (F, G) ROC curve and calibration curve evaluate the predictive accuracy of the nomogram for patient survival at 3, 5, and 7 years. (H) The survival curve shows the prognostic differences between high-risk and low-risk groups based on the nomogram.

Supplementary material 10. Fig. S10. Drug Sensitivity Prediction. (A) IC50 values of sunitinib, temsirolimus, pazopanib, and sorafenib in high-risk and low-risk KIRC patients. (B) IC50 values of sunitinib, temsirolimus, and pazopanib in high-risk and low-risk KIRP patients.

Supplementary material 11. Fig. S11. Immune therapeutic prediction between high-risk and low-risk groups. (A) MCP immune cell infiltration levels in high-risk and low-risk groups. (B) The survival curve demonstrates the prognostic differences between high-risk and low-risk patients in the Alexandra cohort. (C) The ROC curve evaluates the MMCG risk score's predictive accuracy in predicting the patients' prognosis in the Alexandra cohort. (D) Barplot shows the distribution of high-risk and low-risk patients in CR/PR and SD/PD groups. (E) The survival curve demonstrates prognostic differences between high-risk and low-risk patients in the GSE78820 cohort. (F) ROC curve evaluates the predictive accuracy of the MMCGs risk score for prognosis in the GSE78820 cohort. (G) Barplot shows the distribution of high-risk and low-risk patients in CR/PR and SD/PD groups.

Supplementary material 12.

Supplementary material 13.

Supplementary material 14.

## Data Availability

Data sharing is not applicable to this article as no datasets were generated or analysed during the current study.
